# Free-breathing, Contrast Agent–free Whole-Heart MTC-BOOST Imaging: Single-Center Validation Study in Adult Congenital Heart Disease

**DOI:** 10.1148/ryct.220146

**Published:** 2023-02-16

**Authors:** Anastasia Fotaki, Kuberan Pushparajah, Reza Hajhosseiny, Alina Schneider, Harith Alam, Joana Ferreira, Radhouene Neji, Karl P. Kunze, Alessandra Frigiola, René M. Botnar, Claudia Prieto

**Affiliations:** From the Department of Biomedical Engineering, School of Biomedical Engineering and Imaging Sciences, King’s College London, St Thomas’ Hospital, Westminster Bridge Road, Lambeth Wing, 3rd Floor, London SE1 7EH, England (A. Fotaki, K.P., R.H., A.S., J.F., R.N., K.P.K., A. Frigiola, R.M.B., C.P.); Department of MRI in Congenital Heart Disease, Guy’s and St Thomas’ NHS Foundation Trust, London, England (K.P., H.A., A. Frigiola); MR Research Collaborations, Siemens Healthcare Limited, Frimley, England (R.N., K.P.K.); and School of Engineering and Millennium Institute for Intelligent Healthcare Engineering, Pontificia Universidad Católica de Chile, Santiago, Chile (R.M.B., C.P.).

**Keywords:** MR Angiography, Cardiac

## Abstract

**Purpose:**

To assess the clinical performance of the three-dimensional, free-breathing, Magnetization Transfer Contrast Bright-and-black blOOd phase-SensiTive (MTC-BOOST) sequence in adult congenital heart disease (ACHD).

**Materials and Methods:**

In this prospective study, participants with ACHD undergoing cardiac MRI between July 2020 and March 2021 were scanned with the clinical T2-prepared balanced steady-state free precession sequence and proposed MTC-BOOST sequence. Four cardiologists scored their diagnostic confidence on a four-point Likert scale for sequential segmental analysis on images acquired with each sequence. Scan times and diagnostic confidence were compared using the Mann-Whitney test. Coaxial vascular dimensions at three anatomic landmarks were measured, and agreement between the research sequence and the corresponding clinical sequence was assessed with Bland-Altman analysis.

**Results:**

The study included 120 participants (mean age, 33 years ± 13 [SD]; 65 men). The mean acquisition time of the MTC-BOOST sequence was significantly lower compared with that of the conventional clinical sequence (9 minutes ± 2 vs 14 minutes ± 5; *P* < .001). Diagnostic confidence was higher for the MTC-BOOST sequence compared with the clinical sequence (mean, 3.9 ± 0.3 vs 3.4 ± 0.7; *P* < .001). Narrow limits of agreement and mean bias less than 0.08 cm were found between the research and clinical vascular measurements.

**Conclusion:**

The MTC-BOOST sequence provided efficient, high-quality, and contrast agent–free three-dimensional whole-heart imaging in ACHD, with shorter, more predictable acquisition time and improved diagnostic confidence compared with the reference standard clinical sequence.

**Keywords:** MR Angiography, Cardiac

*Supplemental material is available for this article.*

Published under a CC BY 4.0 license

## Graphical abstract

**Figure:**
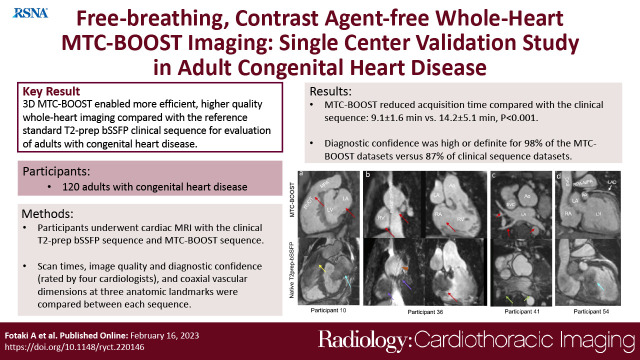

**Summary**

Free-breathing nonrigid motion-compensated whole-heart MTC-BOOST enabled efficient and contrast agent–free three-dimensional anatomic assessment of the heart and thoracic vasculature in participants with adult congenital heart disease.

**Key Points**

■ In a prospective evaluation of 120 participants with congenital heart disease, the Magnetization Transfer Contrast Bright-and-black blOOd phase-SensiTive (MTC-BOOST) sequence reduced the acquisition time for a three-dimensional anatomic cardiac and thoracic data set compared with the clinical native T2-prepared balanced steady-state free precession sequence: 9.1 minutes ± 1.6 (SD) versus 14.2 minutes ± 5.1, *P* < .001.
■ Diagnostic confidence was high or definite for 98% of the MTC-BOOST data sets versus 87% of clinical sequence data sets.
■ Reproducibility analysis showed good agreement for the vascular measurements between the clinical sequence and the MTC-BOOST sequence and excellent intra- and interreviewer agreement for the vascular measurements with MTC-BOOST.

## Introduction

MR angiography is an established sequence for the diagnosis, surveillance, and pre- and postprocedural evaluation of adult congenital heart disease (ACHD) (1,2). For adequate anatomic description, a systematic approach based on sequential chamber and vascular identification and description, namely the sequential segmental analysis, is adopted (3).

Current guidelines suggest the use of T2-prepared balanced steady-state free precession (T2prep-bSSFP) three-dimensional (3D) whole-heart MR angiography (2). However, this sequence is susceptible to off-resonance artifacts in the pulmonary veins, flow-related artifacts due to turbulent blood flow, and metallic artifacts from stents or devices that may impede a full sequential segmental analysis (4,5). Furthermore, the clinical T2prep-bSSFP sequence suffers from long and unpredictable scan times because of the employment of diaphragmatic navigation, leading to acceptance rates as low as 30%–40% in patients with irregular respiratory patterns (6). Younger children and adult patients with congenital heart disease (CHD) experience a slightly higher than average prevalence of developmental delay and disability and may not cooperate well during imaging (7). Therefore, fast acquisition is desirable to foster compliance.

Prior studies have shown that the free-breathing 3D whole-heart Magnetization Transfer Contrast Bright-and-black blOOd phase-SensiTive (MTC-BOOST) sequence for bright-blood imaging and complementary phase-sensitive inversion recovery (PSIR) black-blood imaging provides high luminal signal for both arteries and veins and adequate contrast between myocardium and blood (8–11). The integration with two-dimensional image-based navigation enables 100% respiratory scan efficiency and predictable scan times (12). We hypothesized that the bright-blood MTC-BOOST volume can provide gadolinium-based contrast agent (GBCA)–free 3D whole-heart imaging of all anatomic cardiac segments in participants with ACHD that is higher quality compared with the clinical native T2prep-bSSFP sequence and obtained in shorter acquisition time. Additionally, we hypothesized that the 3D black-blood MTC-BOOST data set can provide supplementary diagnostic information in specific cases, such as in the presence of a thrombus. The aim of this study was to clinically validate the bright-blood MTC-BOOST volume in comparison to the clinical native T2-prep bSSFP sequence and to explore the clinical merits of the black-blood PSIR MTC-BOOST volume.

## Materials and Methods

This prospective cross-sectional cohort study was approved by the National Research Ethics Service (reference no. 15/NS/0030), and written informed consent was obtained from each participant according to institutional guidelines.

Two authors (R.N., K.P.K.) are employees of the industry (Siemens Healthineers) and contributed to the in-line implementation of the research sequence. Only authors who are not industry employees (A. Fotaki, K.P., R.H., and A. Frigiola) had control of the inclusion and analysis of any data and information in this study.

### Study Participants

Patients with ACHD undergoing clinically indicated cardiac MRI from July 2020 through March 2021 at a single tertiary care hospital were consecutively recruited. Exclusion criteria were general MRI exclusion criteria and inability to obtain consent. Patients with epicardial pacemakers were excluded from the study as per departmental research ethics guidelines, along with patients who needed to receive GBCA. Details for sample size calculation are presented in Appendix S1.

### Cardiac MRI Protocol

All acquisitions reported in this study were performed with a 1.5-T system (MAGNETOM Aera; Siemens Healthineers) using an 18-channel chest coil and a 32-channel spine coil. The recruited participants underwent 3D whole-heart imaging with the clinically requested T2prep-bSSFP acquisition and the research MTC-BOOST sequence. The MTC-BOOST sequence was performed at the end of the imaging session, after the clinical protocol.

### MTC-BOOST Sequence

**Figure 1:**
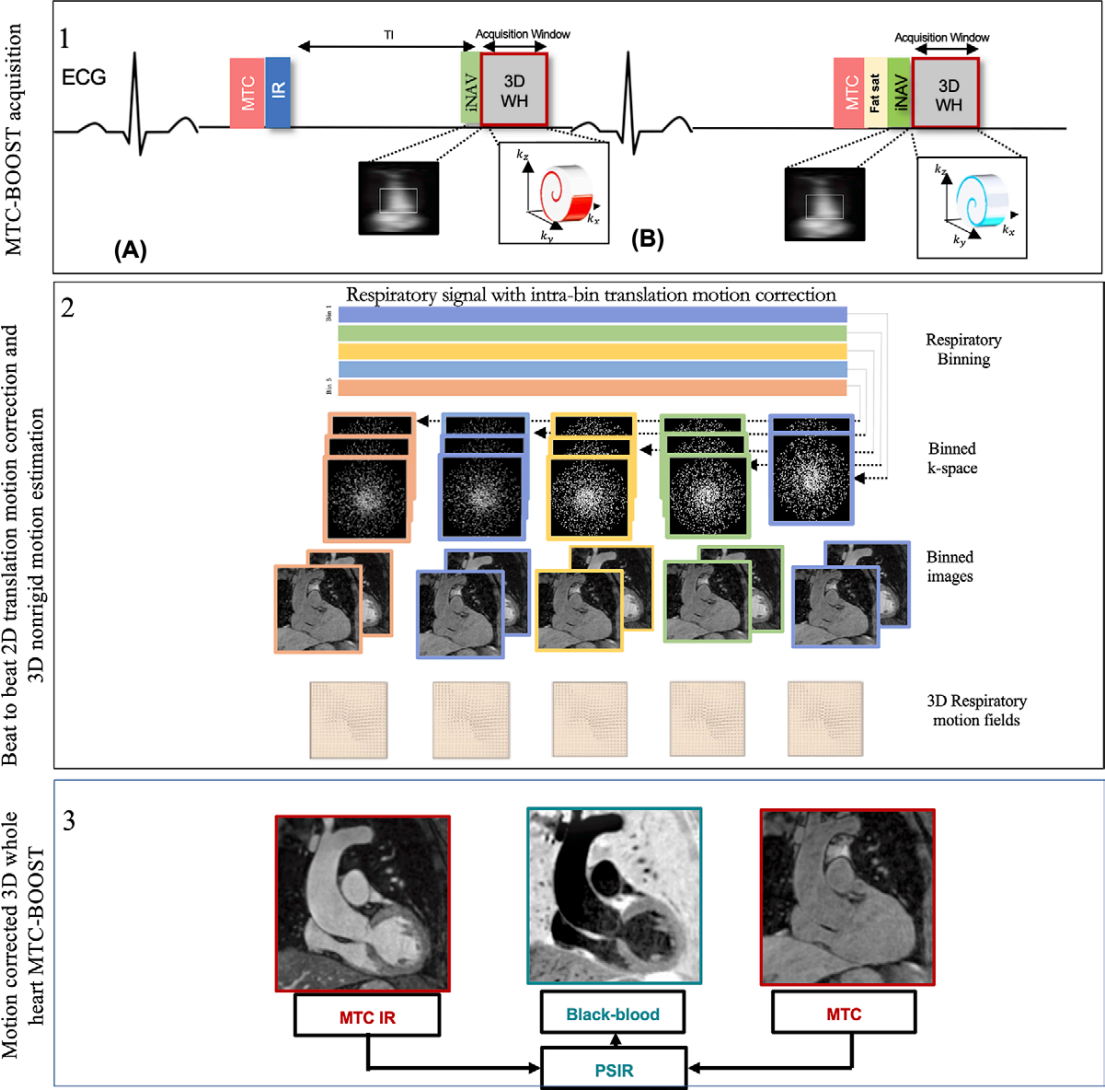
Schematic overview of investigated free-breathing nonrigid motion-corrected three-dimensional (3D) whole-heart MTC-BOOST framework. Top: Two magnetization-prepared bright‐blood volumes are acquired in odd **(A)** and even **(B)** heartbeats. Magnetization transfer in combination with an inversion pulse is used in odd heartbeats, whereas magnetization transfer alone is exploited in even heartbeats. In odd heartbeats, a short inversion time inversion-recovery approach is used to suppress the signal from epicardial fat, whereas frequency‐selective presaturation is used in even heartbeats. Data acquisition is performed using a 3D Cartesian trajectory with spiral profile order. A low‐resolution two-dimensional (2D) iNAV is acquired in each heartbeat by spatially encoding the ramp‐up pulses of the bSSFP sequences. The iNAVs are used to estimate foot-head and right-left rigid motion by tracking a template around the aortic arch, providing motion estimates in a beat-to-beat basis. Middle: Foot-head motion is used to sort the 3D MTC-BOOST data into five equally populated bins, and 3D MR images reconstructed at each respiratory position are used to estimate nonrigid motion between bins. 2D translational beat-to-beat and 3D nonrigid bin-to-bin motion is then integrated into an in-line motion-compensated iterative sensitivity encoding reconstruction to produce the final images. Bottom: The bright‐blood MTC‐IR BOOST and MTC-BOOST volumes are corrected for translation and nonrigid motion and are subsequently combined in a PSIR‐like reconstruction to generate a complementary black‐blood volume. bSSFP = balanced steady-state free precession, ECG = electrocardiography, Fat sat = fat suppression, iNAV = image-based navigator, kx = readout, ky = phase encoding, kz = MRI signal along the scanner bore, MTC-BOOST = Magnetization Transfer Contrast Bright-and-black blOOd phase SensiTive, IR = inversion-recovery pulse, PSIR = phase-sensitive inversion recovery, TI = inversion time, 3D WH = 3D whole-heart.

A free-breathing 3D whole-heart, electrocardiographically triggered, balanced steady-state free precession Cartesian prototype MTC-BOOST sequence (Fig 1), previously described in Ginami et al (9), was performed. Briefly, MTC-BOOST alternates the acquisition of a bright-blood MTC inversion recovery–prepared volume for optimized contrast between blood and myocardium (MTC-IRprep-BOOST, odd heartbeats) (Fig 1, top, A) and a bright-blood MTC-prepared volume (MTCprep-BOOST, even heartbeats) (Fig 1, top, B). A two-dimensional image-based navigator (iNAV) precedes each 3D whole-heart data acquisition to enable beat-to-beat two-dimensional translational respiratory and 3D nonrigid motion estimation and compensation, with 100% scan efficiency (no data rejection) (12,13). The two bright-blood volumes (MTC-IRprep-BOOST and MTCprep-BOOST) are then combined in a PSIR-like reconstruction (11) to generate a complementary black-blood volume, using the MTCprep-BOOST scan (even heartbeats) as the reference image for phase computation. A comprehensive outline of the MTC-BOOST sequence is presented in Appendix S2 and in studies by Ginami et al (8–9) and Rashid et al (10).

Detailed parameters of the clinical sequence and MTC-BOOST sequence are provided in Table S1.

### Qualitative Image Quality Analysis and Diagnostic Confidence

Image processing and reformatting were performed with commercially available analysis software (Horos, version 1.1.7; *https://horosproject.org/*). Two hundred forty data sets were anonymized, de-identified, and randomized for evaluation. Blinded to participant characteristics, four cardiologists who specialize in cardiac MRI for ACHD (reader 1, A. Frigiola; reader 2, K.P.; reader 3, R.H.; and reader 4, A. Fotaki; each with European Association of Cardiovascular Imaging level 3 accreditation and 15, 15, 4, and 3 years of experience, respectively) independently scored the image quality of all intrapericardiac structures. Each reader reviewed 30 separate data sets from each sequence (30 data sets × four readers × two sequences = 240 total data sets). The image quality assessment was based on a five-point scoring system and was divided on sharpness of vessel or cardiac wall borders (from 1 = nondiagnostic to 5 = excellent) and robustness to artifact (from 1 = severe artifact to 5 = minimal artifact). Subsequently, the reviewers scored their diagnostic confidence to perform sequential segmental analysis with each data set using a four-point Likert scale (1 = low confidence; 2 = moderate, but additional imaging required; 3 = high [diagnostic]; 4 = definite). After grading diagnostic confidence, the MRI findings could be adjudicated with locally available echocardiographic, catheterization, CT, and operative data. When these data were not available, independent review by two experts (consultant cardiologists specialized in ACHD and in MRI in ACHD, each with 15 years of experience), blinded to participant information, was obtained. The data anonymization, randomization, and scoring criteria, as based on previous studies (14–16), are detailed in Appendix S3.

### Quantitative Image Quality Analysis

The contrast-to-noise ratio between blood and myocardium was computed in the respective aforementioned structures.

### Measurement Reproducibility Analysis

Vascular dimensions were measured at three landmarks predefined by literature guidelines (17), namely the aortic root and mid ascending and mid right pulmonary arteries. The data sets were anonymized and randomized to prevent comparison between the paired research and clinical data sets and thus to minimize bias. The readers were blinded to the underlying diagnosis and patient demographics. Coaxial measurements (maximum diameter) were performed using multiplanar reformats by two reviewers (K.P. and A. Fotaki), as based on those performed in previous studies (17,18). Reviewers 2 and 4 analyzed 30 separate paired data sets. Measurements were used for comparison between the research and clinical sequences. Reviewer 4 also analyzed the additional sample of 30 paired data sets, which had been previously analyzed by reviewer 2, to assess for intraobserver reliability. Reviewer 4 repeated the measurements in 30 MTC-BOOST bright-blood data sets after a 2-month interval to assess intraobserver reliability.

### PSIR Black-Blood MTC-BOOST Data Sets Analysis

The PSIR black-blood MTC-BOOST data sets were anonymized and reviewed with the corresponding bright-blood MTC-BOOST data sets to investigate whether diagnostic confidence was further enhanced.

### Statistical Analysis

Continuous variables were presented as means ± SDs. In view of the sample size (*n* = 120), normal approximation to the means was adequate due to the central limit theorem. The Student *t* test was used to compare differences in continuous variables between two groups. Subjective scores were compared with a paired Wilcoxon signed rank test to assess statistical differences. Two-tailed values of *P* less than .05 were considered statistically significant differences. The intraclass correlation coefficient was used to assess intra- and interobserver variability; Bland-Altman analysis was used to assess interobserver variability and agreement between the research and clinical methods. Statistical analysis was performed using GraphPad Prism (version 9.1.0; GraphPad Software).

## Results

### Participant Characteristics

**Figure 2:**
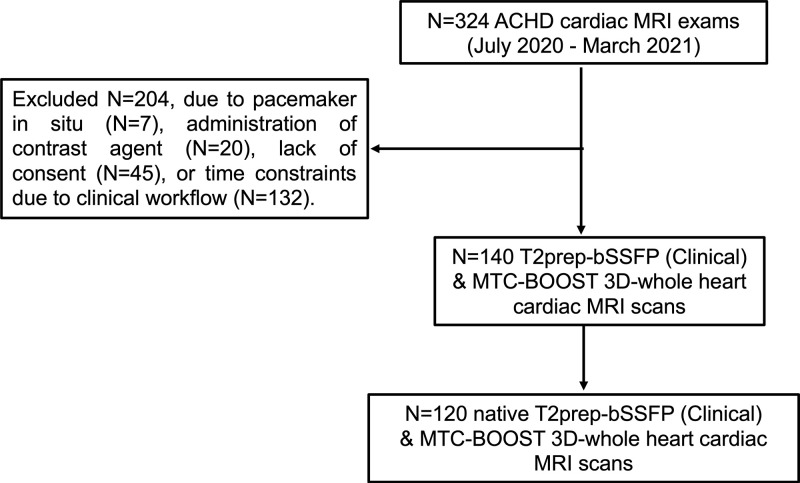
The flowchart outlines the selection of participants with congenital heart disease included in the final analysis of this study. ACHD = adult congenital heart disease, MTC-BOOST = Magnetization Transfer Contrast Bright-and-black blOOd phase SensiTive, T2prep-bSSFP = T2-prepared balanced steady-state free precession, 3D = three-dimensional.

A total of 120 consecutive participants (mean age, 33 years ± 13 [SD]; 65 men) were included in this study and scanned with the clinical T2prep-bSSFP sequence along with the proposed MTC-BOOST sequence (Fig 2). Participant baseline characteristics are listed in Table 1.

**Table 1:**
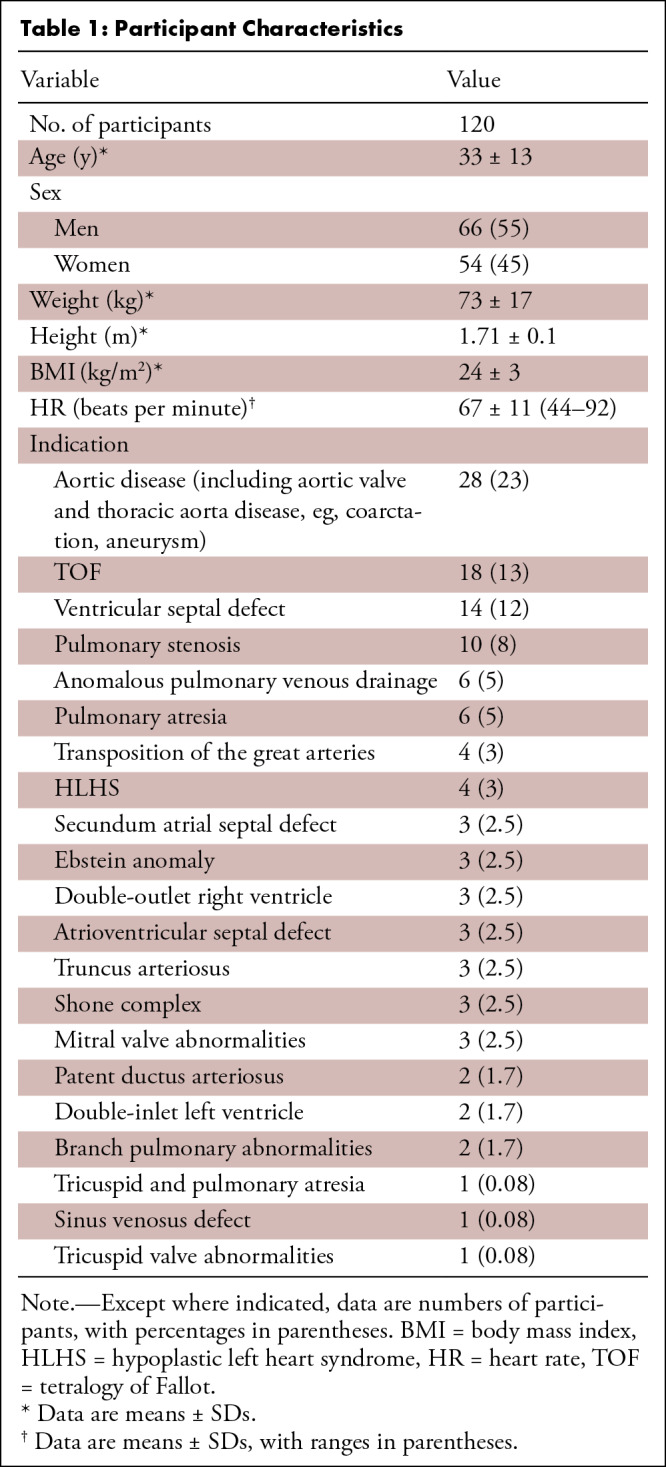
Participant Characteristics

### 3D Whole-Heart MRI Analysis

The acquisition time was statistically significantly lower for the MTC-BOOST sequence versus the T2prep-bSSFP clinical sequence (9.1 minutes ± 1.6 vs 14.2 minutes ± 5.1; *P* < .001).

**Figure 3:**
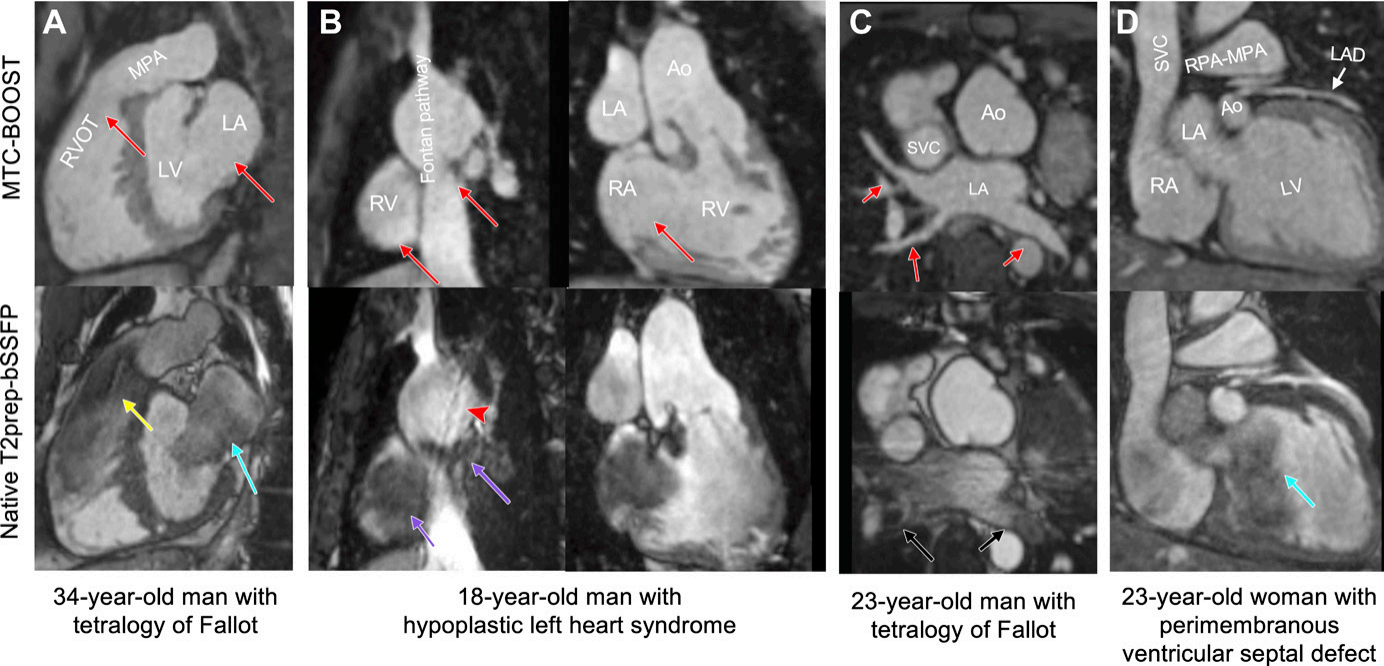
Comparison of MTC-BOOST and native T2prep-bSSFP cardiac MRI. **(A)** Multiplanar reformatted images in a 34-year-old man diagnosed with tetralogy of Fallot after repair with transannular patch. Severe pulmonary artery regurgitation caused signal voids in the right ventricle, right ventricular outflow tract, and main pulmonary artery because of flow artifact (yellow arrow) in the clinical native sequence. Off-resonance artifact is demonstrated in the left atrium (blue arrow). Artifacts are minimized with the proposed MTC-BOOST sequence (red arrows). **(B)** Multiplanar reformatted images in an 18-year-old man with hypoplastic left heart syndrome after total cavopulmonary connection completion with a fenestrated lateral tunnel Fontan pathway. Signal voids are observed in the lateral tunnel and right atrium because of stagnant flow (purple arrows) in the native T2prep-bSSFP clinical data set, which necessitate further imaging for the exclusion of obstruction. Residual respiratory artifact (red arrowhead) is also present. The MTC-BOOST sequence demonstrates the vascular lumen without substantial artifact and excludes obstruction (red arrows). **(C)** Multiplanar reformatted images in a 23-year-old man with tetralogy of Fallot after repair with transannular patch, followed by pulmonary valve replacement with homograft due to severe regurgitation. Off-resonance artifacts in the pulmonary veins in the native T2-prep bSSFP sequence (black arrows) impede the sequential segmental anatomic description. Pulmonary venous return can be established in the MTC-BOOST data set (red arrows). **(D)** Multiplanar reformatted images in a 23-year-old woman with a small perimembranous ventricular septal defect that has not been repaired, causing mild aortic regurgitation. Flow-related artifact in the left ventricle (blue arrow) observed in the clinical native data set is suppressed in the MTC-BOOST data set. The left anterior descending coronary artery is sharply delineated with the research sequence (white arrow), owing to the improved fat suppression. Ao = aorta, LA = left atrium, LAD = left anterior descending artery, LV = left ventricle, MPA = main pulmonary artery, MTC-BOOST = Magnetization Transfer Contrast Bright-and-black blOOd phase SensiTive, RPA = right pulmonary artery, RV = right ventricle, RVOT = RV outflow tract, SVC = superior vena cava, T2prep-bSSFP = T2-prepared balanced steady-state free precession.

**Figure 4:**
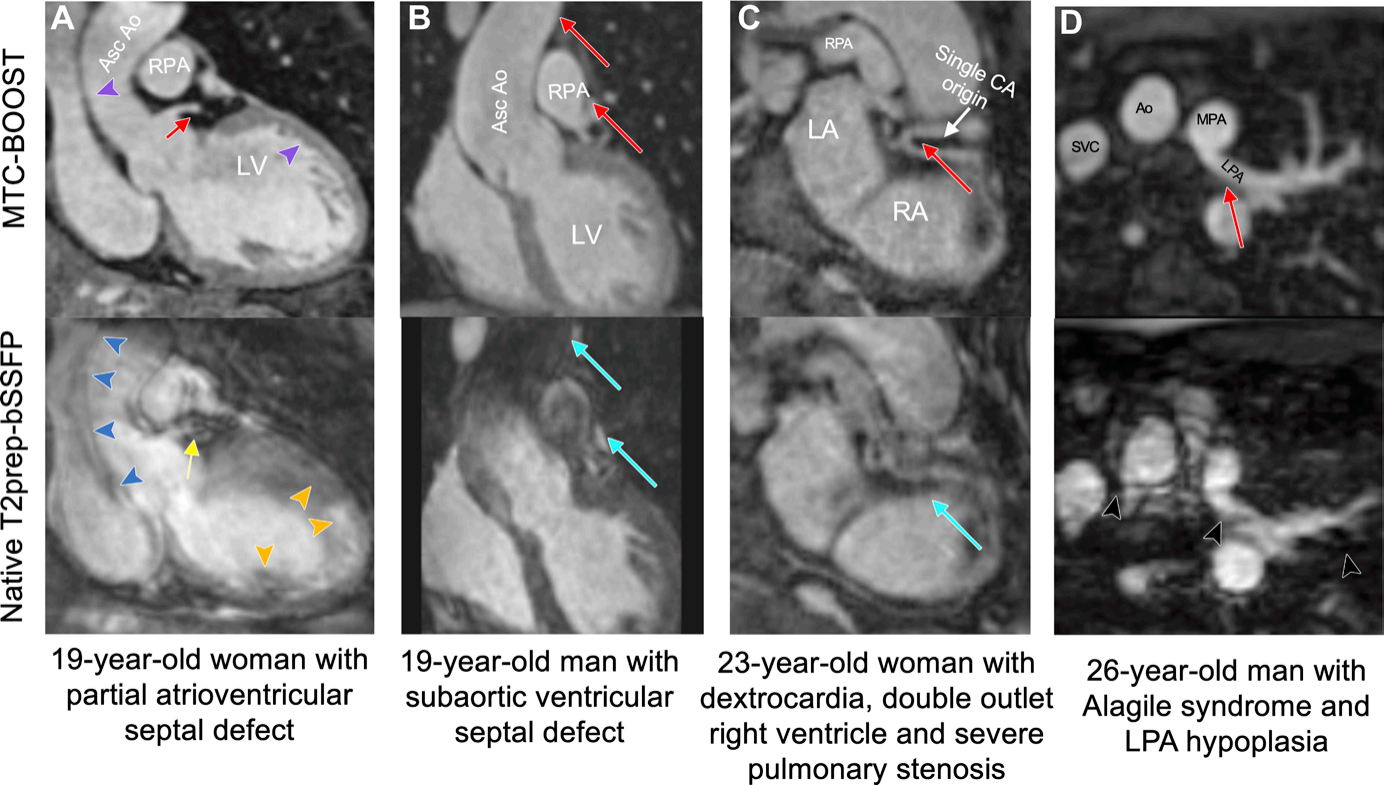
Comparison of MTC-BOOST and native T2prep-bSSFP cardiac MRI. **(A)** Multiplanar reformatted images in a 19-year-old woman with partial atrioventricular septal defect, after repair, and severe left atrioventricular valve regurgitation. Residual respiratory motion induces blurring in the ascending aorta (blue arrowheads), left ventricle (orange arrowheads), and left main stem (yellow arrow) in the clinical T2prep-bSSFP data set. Respiratory motion is adequately resolved in the MTC-BOOST data set, with clear depiction of the aortic and left ventricular wall (purple arrowheads) and the left main coronary stem (red arrow). **(B)** Multiplanar reformatted images in a 19-year-old man with subaortic ventricular septal defect and aortic regurgitation secondary to aortic valve prolapse after surgical repair, who had recurrent aortic regurgitation after ventricular septal defect closure and aortic valve repair. Images were acquired in end systole because of high heart rate. Substantial luminal signal loss in the ascending aorta and right pulmonary artery was observed due to flow artifacts (blue arrows). Attenuation of the artifact in the corresponding regions in the MTC-BOOST data set enabled reliable aortic dimensioning (red arrows). **(C)** Multiplanar reformatted images in a 23-year-old woman with dextrocardia, situs solitus, double-outlet right ventricle, and severe pulmonary stenosis, who was palliated with Hemi-Fontan procedure. Common origin of the coronary arteries from the posterior-facing sinus is well demarcated with the MTC-BOOST sequence (red arrow). Substantial artifact from residual respiratory motion in the clinical T2prep-bSSFP data set (blue arrow) hinders diagnostic certainty. **(D)** Multiplanar reformatted images in a 26-year-old man with Alagile syndrome and hypoplasia of the left pulmonary artery. Residual respiratory motion in the clinical T2prep-bSSFP data set causes substantial blurring along the course of the left pulmonary artery and ascending aorta, leading to unclear measurements of the respective vascular diameters (black arrowheads). The MTC-BOOST data set resolves the respiratory motion and demarcates the aorta, as well as the proximal and distal course of the left pulmonary artery (red arrow) and its branches. Ao = aorta, Asc = ascending, CA = coronary artery, LA = left atrium, LPA = left pulmonary artery, LV = left ventricle, MPA = main pulmonary artery, MTC-BOOST = Magnetization Transfer Contrast Bright-and-black blOOd phase SensiTive, RA = right atrium, RPA = right pulmonary artery, SVC = super vena cava, T2prep-bSSFP = T2-prepared balanced steady-state free precession.

A spectrum of complex morphologic features and vascular anatomy was clearly delineated with MTC-BOOST as shown in Figures 3 and 4. In participants with turbulent flow (Figs 3A, 4B) and low flow or flow stagnation (Fontan pathway) (Fig 3B), the vascular lumen was demonstrated with no signal intensity loss compared with the clinical sequence. The lumen of the pulmonary veins was sharply demarcated, reducing off-resonance artifacts (Fig 3C), and could be traced distally in the lung parenchyma (Fig S1A). Blurring from respiratory motion was attenuated with MTC-BOOST (Figs 3B, 4A, 4C, 4D) with respect to the clinical sequence, resulting in improved vascular dimensioning and coronary delineation.

### Qualitative Image Quality Analysis and Diagnostic Confidence

**Figure 5:**
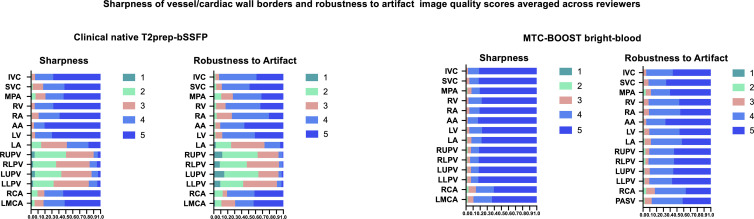
Histogram demonstrating image quality scores for the clinical native T2prep-bSSFP versus bright-blood MTC-BOOST imaging. Summary of the image quality scores with regard to sharpness of vessel or cardiac wall borders and robustness to artifact (120 clinical examinations averaged across four reviewers and 120 research examinations averaged across four reviewers). Vessel sharpness and artifact scoring color correspondence is provided next to the respective color bar. The x-axis reflects percentage of examinations. A maximal image quality score of 5 for vessel sharpness indicates sharp delineation of all relevant anatomic structures with excellent contrast, whereas a robustness-to-artifact score of 5 reflects no ghosting, signal voids, or cardiac motion blurring (Appendix S3). AA = ascending aorta, IVC = inferior vena cava, LA = left atrium, LLPV = left lower pulmonary vein, LMCA = left main coronary artery, LUPV = left upper pulmonary vein, LV = left ventricle, MPA = main pulmonary artery, MTC-BOOST = Magnetization Transfer Contrast Bright-and-black blOOd phase SensiTive, RA = right atrium, RCA = right coronary artery, RUPV = right upper pulmonary vein, RV = right ventricle, SVC = superior vena cava, T2prep-bSSFP = T2-prepared balanced steady-state free precession.

Bright-blood MTC-BOOST yielded vascular sharpness superior to that of the clinical sequence. In delineation of the pulmonary veins (PVs), 95% (114 of 120) of MTC-BOOST examinations had image quality scores of good or excellent versus 15% (18 of 120) of T2prep-bSSFP clinical sequence examinations, with respective scores of 92% (110 of 120) versus 79% (95 of 120) for the main pulmonary artery (MPA), 95% (114 of 120) versus 82% (98 of 120) for the superior vena cava, and 97% (116 of 120) versus 95% (114 of 120) for the ascending aorta. Imaging artifacts were attenuated with MTC-BOOST in all intrapericardiac structures. Grading was performed with minimal artifacts in 90% (108 of 120) versus 8% (10 of 120) of MTC-BOOST and clinical sequence examinations, respectively, for the PVs; 88% (106 of 120) versus 71% (85 of 120) for the MPA; 92% (110 of 120) versus 86% (103 of 120) for the superior vena cava; and 95% (114 of 120) versus 90% (108 of 120) for the ascending aorta. Figure 5 and Table 2 summarize the image quality scores and respective *P* values.

**Table 2:**
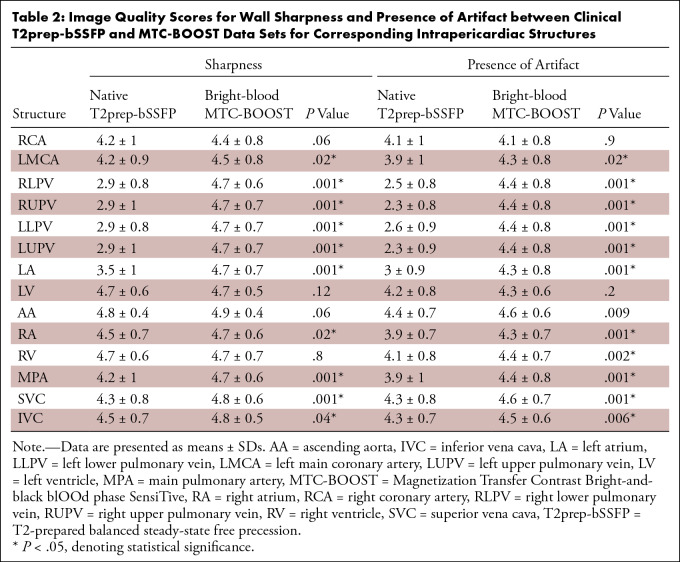
Image Quality Scores for Wall Sharpness and Presence of Artifact between Clinical T2prep-bSSFP and MTC-BOOST Data Sets for Corresponding Intrapericardiac Structures

Diagnostic confidence was higher for MTC-BOOST compared with the clinical sequence (mean, 3.9 ± 0.3 vs 3.4 ± 0.7; *P* < .001) (Table 3). Use of MTC-BOOST achieved full segmental diagnoses in all examinations but two (98% [118 of 120] success rate) in which the PVs could not be visualized because of an artifact from a receiver coil (one case) and an artifact from a metallic aortic valve impeding the depiction of the left main stem (one case). The T2prep-bSSFP clinical sequence failed to provide full sequential segmental analysis in 16 cases (87% [104 of 120] success rate). Failure was due to substantial artifacts in one or more PVs in seven cases, in PVs and the MPA in one case, in PVs and coronary arteries in three cases, in coronary arteries in four cases, and in the MPA and coronary arteries in one case. All 16 diagnoses were confirmed with previous contrast-enhanced T2prep-bSSFP clinical scans in 14 participants, CT in one participant, and cardiac catheterization in the final participant.

**Table 3:**
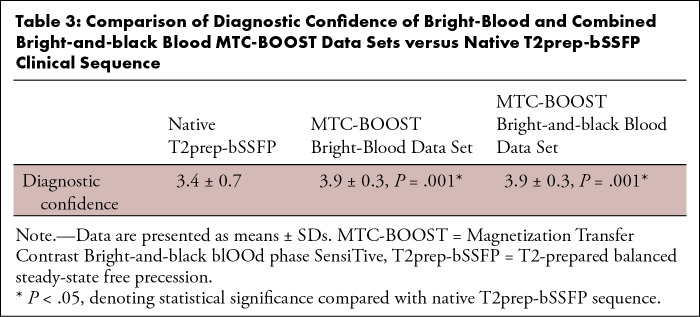
Comparison of Diagnostic Confidence of Bright-Blood and Combined Bright-and-black Blood MTC-BOOST Data Sets versus Native T2prep-bSSFP Clinical Sequence

### Quantitative Image Quality Analysis

The bright-blood MTC-BOOST sequence had higher or comparable contrast-to-noise ratio to the clinical native sequence for all the structures assessed (Table S2).

### Measurement Reproducibility Analysis

**Figure 6:**
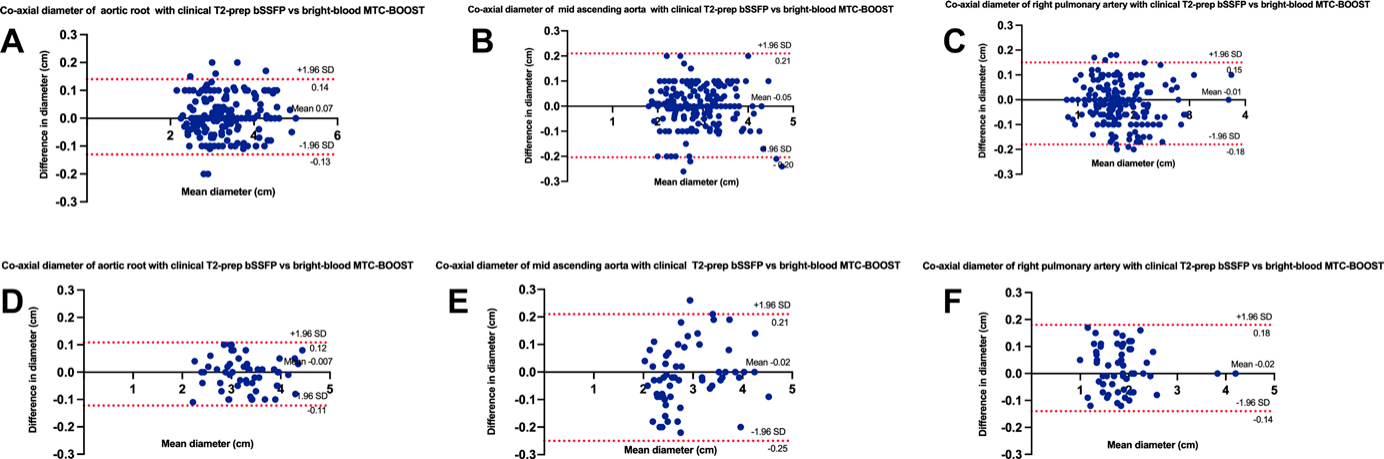
Bland-Altman plots for coaxial diameter measurements of the aortic root, mid ascending aorta, and right pulmonary artery with the clinical native T2prep-bSSFP versus bright-blood MTC-BOOST sequence **(A–C)** for reviewer 4 (A. Fotaki) and **(D–F)** for reviewer 2 (K.P.). The black line indicates the mean bias of the diameter measurements, and the red lines represent the 95% CIs. Values are given in centimeters. Measurements from both reviewers showed good agreement between sequences for all three landmarks. MTC-BOOST = Magnetization Transfer Contrast Bright-and-black blOOd phase SensiTive, T2-prep bSSFP = T2-prepared balanced steady-state free precession.

There was excellent agreement between the coaxial diameter measurements for each sequence. Compared with T2prep-bSSFP measurements, MTC-BOOST measurements at the defined landmarks had a mean difference of less than 0.07 cm and narrow limits of agreements for both reviewers (Fig 6).

Intraclass correlation coefficients for intra- and interobserver agreement analysis for each rater are summarized in Table S3. The mean difference between the inter- and intraobserver measurements at the defined landmarks with the proposed MTC-BOOST sequence was less than 0.03 cm (Fig S2).

### PSIR Black-Blood MTC-BOOST Data Sets Analysis

There were two participants with subaortic fibromuscular ridge whose morphologic features were more evident in the black-blood MTC-BOOST data set (Fig S3A). The black-blood MTC-BOOST data set showed the thrombus in one participant (Fig S1B). These findings were confirmed intraoperatively and with CT imaging, respectively.

Black-blood MTC-BOOST imaging did not null the signal from stents or devices. Although signal voids in the immediate vicinity of the device were common, substantial disruption of surrounding tissue was not observed when compared with the clinical sequence (Fig S3B–D).

Despite the three discrete cases for which black-blood MTC-BOOST offered particular diagnostic advantage, the overall diagnostic confidence did not change when the black-blood MTC-BOOST data set was reviewed in addition to the bright-blood MTC-BOOST data set, compared with the clinical T2prep-bSSFP sequence (median, 4 [IQR, 4–4] vs 4 [IQR, 3–4]; *P* = .001) (Table S4).

## Discussion

In this study, we investigated the clinical performance of a prototype MTC-BOOST sequence, which adopts endogenous magnetization transfer contrast and inversion recovery preparation with iNAV for respiratory and nonrigid motion correction, for assessment of the cardiac anatomy and the thoracic vasculature in 120 participants with ACHD. The findings can be summarized as follows: The proposed GBCA-free and free-breathing acquisition was performed in significantly faster and more predictable time than the reference standard clinical sequence (9 minutes ± 2 vs 14 minutes ± 6; *P* < .001); it achieved higher overall diagnostic confidence than the conventional sequence (*P* < .001); and it produced reliable quantification of the vascular dimensions, with excellent intra- and interobserver agreement.

Several factors contribute to robust image quality scores for the proposed framework. In-line translational and nonrigid motion correction with iNAV enables 100% respiratory scan efficiency, along with improved suppression of motion-induced artifacts. Additionally, the magnetization transfer contrast preparation pulse is less sensitive to flow artifacts compared with the T2prep module, which relies on refocusing the transverse magnetization and suffers from flow-related signal loss in the case of turbulent flow (2,5). This is particularly frequent in anatomic imaging of ACHD (stenotic vessels, regurgitant jets, calcified homografts). Similarly, the proposed MTC-BOOST sequence offers uniform contrast across all cardiac chambers and vessels, whereas the T2prep sequence is susceptible to the paramagnetic effects of deoxyhemoglobin, causing variations in the magnetic field and artifacts in images of the systemic veins (19). This can be detrimental in imaging of the Fontan pathway and venous baffles in patients with transposition of the great arteries after Mustard and Senning procedures, where signal voids due to stagnant flow cannot be easily distinguished from suspected thrombus, necessitating further imaging (20). The reduction in luminal signal observed in PVs with conventional T2prep-bSSFP imaging is likely mediated by off-resonance effects as previously demonstrated by Hu et al (4). In contrast, the bright-blood MTC-BOOST sequence yielded clear and consistent delineation of PVs associated with substantially higher image quality scores and corresponding increase in luminal contrast ratio.

Spin-echo black-blood imaging is used in combination with T2prep-bSSFP because it is relatively robust to flow-related and off-resonance artifacts and artifacts from devices, depending on the thickness, direction, and velocity of flow (21). MTC-BOOST (bright-blood) obviates the requirement for this sequence when the former two factors are present. The MTC-BOOST black-blood data set is primarily T1 weighted and thus demonstrated to be promising for the delineation of thrombus and thin-walled structures, including subaortic fibromuscular ridges (8,9). However, the bright-blood and black-blood MTC-BOOST data sets cannot demonstrate vascular patency in the presence of stents or ferromagnetic devices with absolute certainty. Spin echo may not be the optimal means either, as the degree of stenosis, if any, cannot be quantified (21).

A recent study has reviewed a 3D fast spin-echo sequence for full segmental diagnoses in a cohort with CHD and reported a success rate of 24%, rising to 100% when combining both contrast-enhanced T2prep-bSSFP clinical and fast spin-echo sequences (22). In our cohort, a similar score was achieved with the single native MTC-BOOST sequence. A recent multicenter study in pediatric patients with CHD proposed the use of feromuxytol-enhanced four-dimensional multiphase steady-state imaging with fast acquisition time to dynamically capture all anatomic features (23). However, off-label use of feromuxytol in MRI is currently approved only in the United States. Additionally, this study was limited to pediatric patients with CHD who underwent MRI under general anesthesia, and the sequence is reliant on the regularity of the respiratory waveform, using the airway pressure signal for respiratory gating. As an inherently native sequence acquired under self-ventilatory free-breathing status (no contrast material needed and no patient cannulation or patient ventilation required), the proposed 3D whole-heart MTC-BOOST sequence reduces scan time, preparation time, and complexity.

Previous studies implementing an initial version of the MTC-BOOST framework, which involved anisotropic acquisition (8,10,24) and only translational respiratory motion correction (8,10,24), demonstrated that it is suitable for PV depiction and has potential for flow-related artifact reduction (10,24). The current study has applied an isotropic MTC-BOOST sequence and considered translational and nonrigid motion correction, along with in-line reconstruction in the imager. Additionally, the current framework was validated in a large and anatomically diverse sample of participants with ACHD. State-of-the-art morphologic assessment in CHD, as proposed by Van Praagh and by Shinebourne et al (3), is challenging with the current resolution for both the proposed and the clinical sequence. For example, straddling atrioventricular valves (chordae) in the mitral valve apparatus are hard to assess. Nevertheless, we believe the MTC-BOOST sequence, with its resistance to flow- and off resonance–related artifacts and its excellent definition of pulmonary venous anatomy, lends itself for this role. Once combined with further acceleration strategies, potentially incorporating deep learning, the sequence may be able to achieve a resolution of 1 mm (24–26).

Our study had limitations. First, this was a single-center study. Further assessment of precision, through reproducibility studies between vendors and sites that include pediatric patients, should be investigated. The integration of arrhythmia detection and rejection algorithms that could help prevent residual cardiac motion artifacts should also be evaluated in future work. Additionally, due to research time constraints mandated by the institutional ethics board, comparisons with alternative whole-heart black-blood techniques and contrast-enhanced sequences were not performed and should be investigated in future work. Furthermore, the sequence type can be inferred from the images (coronal orientation for the MTC-BOOST sequence vs sagittal for the clinical sequence); therefore, despite the anonymization and de-identification of the data sets, fully blinded evaluations were not feasible. However, the data sets were randomized between the readers to minimize bias.

In conclusion, the MTC-BOOST sequence achieved 3D whole-heart bright-blood imaging in participants with ACHD, with robust image quality. The proposed approach mitigated frequently encountered artifacts (off-resonance, flow, respiratory and device related) and demonstrated potential for visualization of the thrombus through a complementary dark-blood data set. The MTC-BOOST sequence achieved higher diagnostic confidence compared with the clinical sequence in a diverse study sample, with the additional benefit of shorter acquisition times. Future multicenter studies across all age groups are required to demonstrate its suitability for reliable, efficient, and contrast agent–free anatomic imaging in CHD.
